# What Foreigners Think of Us

**Published:** 1852-07

**Authors:** 


*What Foreigners think of us.*

Among the various reasons urged in favor of changing the constitution of the American Medical Association, that most weighty with many was the fact that its "Transactions" had been condemned by certain foreign reviewers. Without enquiring whether any change would be likely to render the "Transactions" more acceptable to those transatlantic critics, we beg simply to state our reasons for believing that such opinions should have no weight whatever, nor influence in the slightest degree the action of the American Medical Profession.

The first of these reasons is, that European doctrines received and adopted too exclusively, have hitherto been the bane of the profession in this country. Without embracing under this head homœopathy, hydropathy, and other doctrines which the profession is unanimous in stigmatizing as quackery, and which have been imported and supported by foreign influence, it is obvious that the practice of excessive medication, more destructive by far to our true interests than both the former, has sprung from the same source. The doctrine of universal purgation promulgated by Hamilton, practiced by Abernethy with his blue pill and black draught, (infusion of senna,) carried out as it has been in this country in the indiscriminate use of calomel, jalap, colocynth, rhubarb, salts, etc., etc., etc., simple, combined, or in tinctures, elixirs, Gregory's and other pills, either manufactured by horse or steam power or by hand, as the "thunder and lightning pills" of "heroic" practitioners, and applied to the diseases of men and women, children and the aged, fevers, inflammations, dropsies and consumptions, diarrhœas and constipation, malarious and nervous and all other diseases, is but one sample of the effects of foreign influence.

Brousaisism, a pathology retrograde beyond that of Hippocrites, and resulting in a practice totally inert, was of the same source.

The numerous hobby horses ridden with more or less eclat by physicians in various parts of the land, such as spinal irritation, irritation of the os uteri, pessaries, &c., are minor specimens from the same source.

Let it not be said that these have passed away and a truer philosophy of medicine taken their place.

The anatomical school, which is essentially that reigning at the present time, rich as it is in the details of morbid structure, and essential as its facts are to science, is yet essentially wanting in truth and comprehensiveness as a system. Its deductions, in a practical sense, are erroneous; it is impossible it should be otherwise. It has been correctly characterized as a "meditation upon death."

All Louis' boasted researches on phthisis did not enable him to do more than pronounce the disease incurable, and are even less serviceable than the discovery of the use of cod liver oil. The same might be said of his work on Typhus.

One branch of the anatomical school is that devoted more especially to microscopic investigations, and while we hail with a just pleasure the facts thus recorded, we should be cautious not to regard it as more than a subordinate means of advancing medical science. What single practical deduction has it yet afforded?

Animal chemistry is the most recent development of this school. Its practical results have been nothing better than those of the strictly anatomical studies. Like them it has corrected errors, revealed some facts, but is essentially impotent to form a basis of medical practice.

These form, in their various details and ramifications, the science and the literature of modern medicine. Whatever conforms to it will be praised by foreign reviewers, whatever dissents from it will be condemned. They cannot conceive of a medical doctrine and philosophy different from their own—higher and truer. Such there must be—such, indeed, there is to some extent at present.

When the morbid anatomy of the present day, the chemistry of the solids and fluids, the cells and nuclei, shall have assumed, as they must, the position of subordinate parts in medical science, the use of ergot in obstetrics, of anæsthetics in pain and spasm, both American discoveries, will remain as final results—triumphs of the science. They were attained by the only true system—experiment on the living *human* body.

There is a practice already gaining ground, established, indeed, to a great extent, particularly in the Western States, taking the place of the purgative, bleeding, starving system,—which, as a system, is American and, unprotected by any great name, is due to the general sense and observation of the profession. It consists in the administration of full doses of opium with quinine and diaphoretics in the first stage of most acute inflammations, fevers and injuries likely to be followed by inflammation.

Those who were so much surprised at the results of the late U. S. census in regard to the ratio of mortality in different States, should not neglect this point. It is the influence of medical practice which makes the difference.

We are to seek then a higher and truer philosophy of medicine than the present. Distrusting the daily discoveries of the microscope and morbid anatomy as bases of practice, we are to extend our views over a wider space of time and regard man from a higher and more universal point of view.

This system of true medical philosophy, where is it more likely to find development than in America? Why not look to nature and the unbiassed observation of American physicians rather than to the past, and a few foreign professors and editors for advancement?

Have not the proceedings of Congress, our social and political systems, been condemned by "European reviewers" because they differed from their own, but were more true. They have developed themselves rapidly and surely, notwithstanding all the sneers cast upon them. So will it be with medical science when physicians come together to exchange views on questions of science and practice, instead of discussing personal interests, the interests of sects and of schools,—when they no longer look to what "they say of us" in Europe.

To the West more especially do we look for that movement which shall tend to a national medical character. Held subserviant, as it has heretofore been, to the East, as the East to Europe, for its books and doctrines, there are recent symptoms that it will soon have opinions of its own. Dr. Dowler has shown what might be done in slaying European monsters by one possessed of the independence to think and the freedom of acting without the fear of foreign or American reviewers before his eyes. Let us hope that his example will not be lost; that individually and collectively the profession will march forward to the discovery of those new parts of the science upon which its future rank essentially depends.

